# Microbial Translocation Is Linked to a Specific Immune Activation Profile in HIV-1-Infected Adults With Suppressed Viremia

**DOI:** 10.3389/fimmu.2019.02185

**Published:** 2019-09-13

**Authors:** Mehwish Younas, Christina Psomas, Christelle Reynes, Renaud Cezar, Lucy Kundura, Pierre Portales, Corinne Merle, Nadine Atoui, Céline Fernandez, Vincent Le Moing, Claudine Barbuat, Olivier Moranne, Albert Sotto, Robert Sabatier, Pascale Fabbro, Thierry Vincent, Catherine Dunyach-Remy, Audrey Winter, Jacques Reynes, Jean-Philippe Lavigne, Pierre Corbeau

**Affiliations:** ^1^Institute of Human Genetics, CNRS-Montpellier University, UMR9002, Montpellier, France; ^2^Infectious Diseases Department, University Hospital, Montpellier, France; ^3^Institute for Functional Genomics, Montpellier University, UMR5203, Montpellier, France; ^4^Immunology Department, University Hospital, Nîmes, France; ^5^Immunology Department, University Hospital, Montpellier, France; ^6^IRD UMI 233, INSERM U1175, Montpellier University, Montpellier, France; ^7^Montpellier University, Montpellier, France; ^8^Infectious Diseases Department, University Hospital, Nîmes, France; ^9^Nephrology Department, University Hospital, Nîmes, France; ^10^Medical Informatics Department, University Hospital, Nîmes, France; ^11^U1047, INSERM, Microbiology University Hospital Nîmes, Montpellier University, Nîmes, France

**Keywords:** bacterial translocation, cell activation, inflammation, coagulation, endothelium

## Abstract

Persistent immune activation in virologically suppressed HIV-1 patients, which may be the consequence of various factors including microbial translocation, is a major cause of comorbidities. We have previously shown that different profiles of immune activation may be distinguished in virological responders. Here, we tested the hypothesis that a particular profile might be the consequence of microbial translocation. To this aim, we measured 64 soluble and cell surface markers of inflammation and CD4+ and CD8+ T-cell, B cell, monocyte, NK cell, and endothelial activation in 140 adults under efficient antiretroviral therapy, and classified patients and markers using a double hierarchical clustering analysis. We also measured the plasma levels of the microbial translocation markers bacterial DNA, lipopolysaccharide binding protein (LBP), intestinal-fatty acid binding protein, and soluble CD14. We identified five different immune activation profiles. Patients with an immune activation profile characterized by a high percentage of CD38+CD8+ T-cells and a high level of the endothelial activation marker soluble Thrombomodulin, presented with higher LBP mean (± SEM) concentrations (33.3 ± 1.7 vs. 28.7 ± 0.9 μg/mL, *p* = 0.025) than patients with other profiles. Our data are consistent with the hypothesis that the immune activation profiles we described are the result of different etiological factors. We propose a model, where particular causes of immune activation, as microbial translocation, drive particular immune activation profiles responsible for particular comorbidities.

## Introduction

Immune activation plays a major role during HIV-1 infection. In the short term, it is the main driver of CD4 T-cell loss (1). In the long term, it fuels non-AIDS-linked morbidities such as atherothrombosis, osteoporosis, metabolic syndrome, neurocognitive disorders, liver steatosis, kidney failure, frailty, and certain types of cancer, even under antiretroviral therapy (ART) (2). These diseases are responsible for over 80% of deaths among virologic responders (3). It is therefore of major interest to better understand the causes and the phenotypes of this immune activation, as well as the pathophysiological mechanisms resulting in its consequences.

Via cell-surface and soluble markers, we recently analyzed the state of activation of the immune system in 120 efficiently treated HIV-1 patients. Using two independent hierarchical clustering analyses, we identified five patient groups characterized by very different immune activation profiles (4). This observation highlights the fact that virological responders do not all present with the same types of immune activation. It is notable that one of these five immune activation profiles was strongly linked to insulin resistance (Odds Ratio 17.06 [95% CI 2.14–135.60], *p* = 0.007). These findings provoke the interesting possibility that certain immune activation profiles might lead to specific comorbidities. It is also logical to hypothesize that these immune activation profiles might be the consequences of specific causes. Various causes of immune activation have been unveiled, including residual viral production, coinfections, CD4 T-cell lymphopenia, immune senescence, metabolism disorders, CD4 T-cell subset deregulation, and microbial translocation (5). In a given virologic responder, some of these causes may prevail. For instance, microbial translocation is present in some individuals but not in others (6). Likewise, some patients have restored their CD4 count, whereas others have not. In addition, the level of persistent HIV production is also variable among virologic responders.

Microbial translocation is the passage of microbes and/or microbial products from the gut lumen into the organism in absence of bacteremia. It has been recognized as a potential source of immune activation in HIV infection (7). It is caused by the conjunction of physical destruction (8) and loose junctions (9) of the epithelium and immunological (CD4 T-cell, and particularly Th17 lymphopenia (10), neutrophil accumulation) (11) lesions of the gut mucosa. It may be noted that some degree of mucosa alteration and microbial translocation may persist under efficient ART (6). Various biomarkers are used to evaluate microbial translocation. Lipopolysaccharide (LPS) and bacterial DNA peripheral blood concentrations are direct markers. LPS-binding protein (LBP) (12) and soluble CD14 (sCD14), the production of which is induced by the presence of LPS, are indirect markers. Intestinal fatty acid binding protein (I-FABP), released by damaged gut epithelial cells, is a marker of epithelium destruction (13). More recently, dysbiosis, an imbalance in bacterial taxa, has been described in the microbiota of HIV patients, linked to microbial translocation and immune activation (14–16). Yet, this dysbiosis might be more linked to sexual preference than to HIV infection (17), and its role as a cause and/or a consequence of immune dysregulation remains yet to be clarified.

In the present study, to test the hypothesis that some causes of immune activation might specifically fuel certain profiles of immune activation, we looked for a link between microbial translocation and the immune activation profiles that we identified in virologic responders.

## Materials and Methods

### Study Design

In the previous ACTIVIH trial, we had analyzed 120 HIV-1 patients over 45 years of age with pre-therapeutic CD4 cell counts below 350 cells per μL. In order to diversify our study population, we recruited 20 additional adults infected by HIV-1 without age or CD4 nadir limitations. All individuals were aviremic (<50 copies per mL) for at least 6 months while under stable antiretroviral regimen. Pregnant or breastfeeding women, persons under immunomodulatory treatment or presenting with diseases likely to modify their immune system were not included. Fourty-seven HIV-negative control subjects matched for age were also recruited. This study was approved by the Ethics Committee of Montpellier University Hospital. All patients had provided written informed consent. The trial was registered on ClinicalTrials.gov under the reference NCT02334943.

### Quantification of Cell Surface and Soluble Markers

Cell surface markers were analyzed by flow cytometry as previously described (4). Soluble markers of immune, endothelial, and coagulation activation were analyzed as in the ACTIVIH study (4). The 64 markers are listed in Supplementary Table 1 (4). 16s ribosomal DNA (rDNA) was quantified by PCR (18). LBP (Enzo Life Sciences) and I-FABP (Hycult Biotech) were measured in plasma using commercial ELISA kits.

### Statistical Analysis

We used the squared Euclidean distance of scaled data to measure dissimilarities between patients and the square of (1-(pearson correlation coefficient)^∧^2) to measure dissimilarities between markers. The classifications used the Ward method (on the squared distance) as a metric. The heatmap was generated using R software. We tested different criteria to choose the number of clusters, and finally chose five clusters as it was consistent with the dendrogram structure and gave homogenous and distinct profiles. We used the Mann-Whitney test to compare patients and controls, and Profile D with other profiles. The links between biomarkers were determined by Spearman rank correlations.

## Results

### Study Subjects

Compared with the first 120 patients we recruited for the ACTIVIH study, the 20 new patients were younger (50.4 ± 12.9 vs. 56.5 ± 8.1 years of mean ± SD age, *p* = 0.054), and presented with shorter durations of HIV infection (8.9 ± 7.1 vs. 17.2 ± 7.4 years, *p* = 0.004), shorter durations of aviremia (56 ± 40 vs. 102 ± 47 months, *p* < 0.001), and a mean pre-therapeutic CD4 cell count tending to be higher (239 ± 169 vs. 192 ± 108 cells per μL, *p* = 0.112). The bioclinical characteristics of all 140 patients and of HIV-negative controls are given in Table 1.

**Table 1:** Bioclinical characteristics of the study populations.

**Characteristic**	**Variable**	**Patients**	**Controls**
Number of individuals		140	47
Age (years)	Mean (± SD)	56 (± 9)	56 (± 13)
% CD4+ T cell	Mean (± SD)	49.3 (± 11.4)	NA
CD4 Count (cell/mm3)	Mean (± SD)	733 (± 375)	NA
CD4/CD8 Ratio	Mean (± SD)	1.24 (± 0.88)	NA
Pre-therapeutic nadir CD4 count (cells/μL)	Mean (± SD)	199 (± 119)	NA
Pre-therapeutic viremia (RNA copies/ml)	Mean (± SD)	1437216 (± 9602969)	NA
Years of HIV infection	Mean (± SD)	16 (± 8)	NA
Months of viral suppression	Mean (± SD)	95 (± 49)	NA
Male sex (%)	*N* (%)	113 (81)	23 (50)

### Immune Activation Profiling

As previously performed, for the 20 additional patients living with HIV-1, we determined the proportions of CD4+ and CD8+ T-cells, naïve, central memory, and effector memory T-cells, based on CD45RA and CD27 expression, activated (HLA-DR+ and/or CD38+), exhausted (PD-1+), and senescent (CD57+, eventually CD27– and CD28–) T-cells. The percentages of activated (HLA-DR+), dysfunctional (CD56–), and senescent (CD57+) NK cells were also measured. Immunoglobulin (Ig)G, IgA, IgM, and soluble CD163 (sCD163) peripheral blood levels were used as markers of B-cell and monocyte activation, respectively. It should be noted that sCD14 was not used as an activation marker, but as a microbial translocation marker. Inflammation was evaluated via soluble Tumor Necrosis Factor receptor type I (sTNFRI-1) and C-reactive protein (CRP) concentrations, and endothelium activation was evaluated via soluble Endothelial Protein C Receptor (sEPCR), soluble Thrombomodulin (sThrombomodulin), and tissue Plasminogen Activator (tPA) concentrations in peripheral blood.

**Figure 1:**
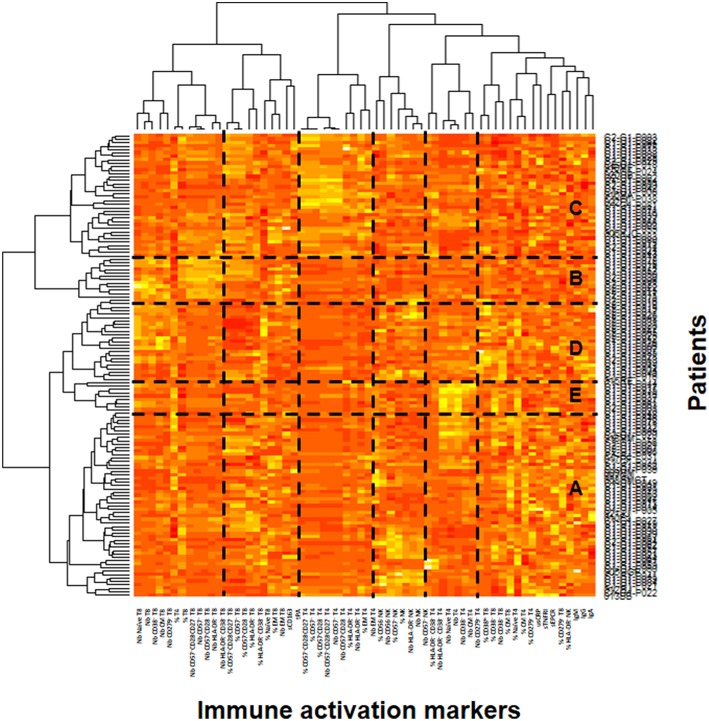
Identification of the patient's immune activation profiles. Heatmap showing the hierarchical clustering of the activation markers (vertical) and of the patients (horizontal). The five profiles of immune activation issued from the patients clustering **(A–E)** are indicated.

**Figure 2:**
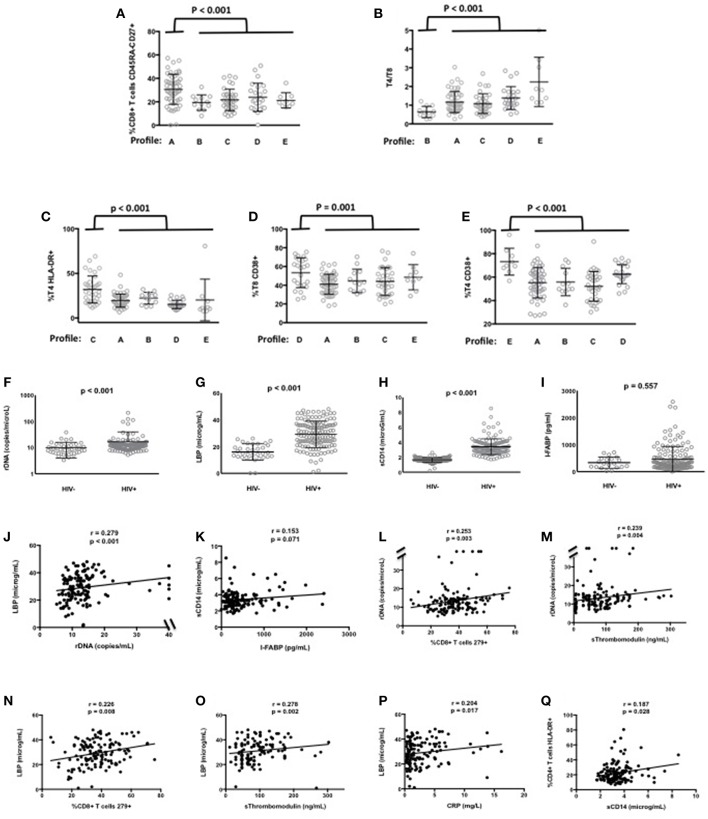
Characterization of the immune activation profiles **(A–E)**. Differences in the level of various activation markers between each cluster of patients and the other clusters are shown. Microbial translocation in patients and healthy donors **(F–K)**. Plasma levels of rDNA **(F)**, LBP **(G)**, sCD14 **(H)**, and I-FABP **(I)** are presented as mean values and standard deviation; *p*-values are shown. Correlations between rDNA and LBP **(J)**, and between sCD14 and I-FABP **(K)** in patients are shown. Correlations between microbial translocation and immune activation markers in patients **(L–Q)**.

In the ACTIVIH study, two independent hierarchical clustering analyses of the activation markers and of the 120 patients had identified five groups of individuals presenting with very different profiles of immune activation. We carried out the same analysis on all 140 patients (Figure 1). Interestingly, although the 20 additional patients presented with divergent bioclinical characteristics, this new analysis again identified five different immune activation profiles. The bioclinical characteristics of the patients are given according to their immune activation profile in Table 2. Each profile may be characterized by a specific marker. As compared with the other patients, patients with Profile A present with a high percentage of central memory CD8+ T-cells (31 ± 13 vs. 22 ± 9%, *p* < 0.001, Figure 2A), and patients with Profile B present with the lowest CD4:CD8 ratio (0.64 ± 0.30 vs. 1.29 ± 0.90, *p* < 0.001, Figure 2B). Profile C patients have the higher percentage of HLA-DR+ CD4+ T-cells (32 ± 15 vs. 19 ± 10%, *p* < 0.001, Figure 2C), whereas Profile D patients have the higher percentage of CD8+ T-cells expressing CD38 (53 ± 3 vs. 43 ± 1%, *p* = 0.001, Figure 2D). Finally, Profile E is remarkable for its elevated proportion of CD38-positive CD4+ T-cells (73 ± 11 vs. 56 ± 12%, *p* < 0.001, Figure 2E).

**Table 2:** Bioclinical characteristics of the participants according to their immune activation profile.

**Characteristic**	**Variable**	**Profile A**	**Profile B**	**Profile C**	**Profile D**	**Profile E**
Number of individuals		56	13	37	25	9
Age (years)	Mean (± SD)	57 (± 9)	57 (± 5)	59 (± 11)	52 (± 7)	54 (± 4)
% CD4+ T cell	Mean (± SD)	48.8 (± 11.7)	36.5 (± 10.7)	47.3 (± 10.8)	53.2 (± 9.6)	63.4 (± 11.6)
CD4 Count (cell/mm^3^)	Mean (± SD)	606 (± 279)	800 (± 344)	678 (± 280)	731 (± 301)	1567 (± 451)
CD4/CD8 Ratio	Mean (± SD)	1.17 (± 0.57)	0.64 (± 0.30)	1.08 (± 0.53)	1.38 (± 0.61)	2.64 (± 2.37)
Pre-therapeutic nadir CD4 count (cells/μL)	Mean (± SD)	148 (± 112)	147 (± 83)	180 (± 98)	148 (± 108)	201 (± 93)
Pre-therapeutic viremia (RNA copies/ml)	Mean (± SD)	2,774,841 (±15,256,275)	148,346 (± 151,297)	492,278 (± 1,835,761)	1,130,295 (± 2,941,405)	447,255 (± 503,358)
Years of HIV infection	Mean (± SD)	15 (± 7)	20 (± 6)	17 (± 8)	14 (± 9)	16 (± 7)
Months of viral suppression	Mean (± SD)	96 (± 44)	81 (± 43)	114 (± 58)	85 (± 45)	99 (± 44)
Male sex	*N* (%)	48 (85)	12 (92)	31 (83)	18 (71)	5 (55)

### Microbial Translocation Markers

Next, for these 140 patients we measured peripheral blood mean (±SEM) levels of rDNA, LBP, sCD14, and I-FABP. Plasma concentrations of rDNA (16.6 ± 2.0 vs. 9.9 ± 0.9 copies/μL, *p* < 0.001, Figure 2F), LBP (29.2 ± 0.8 vs. 16.0 ± 1.0 μg/mL, *p* < 0.001, Figure 2G), and sCD14 (3.41 ± 0.09 vs. 1.65 ± 0.02 μg/mL, *p* < 0.001, Figure 2H) were significantly higher, whereas I-FABP concentrations (470 ± 39 vs. 333 ± 46 pg/mL, *p* = 0.557, Figure 2I) were non-significantly higher in HIV patients than in HIV-negative controls matched for age. In our search for correlations between microbial translocation markers, we observed a link between rDNA and LBP (*r* = 0.279, *p* < 0.001, Figure 2J), and an almost significant link between sCD14 and I-FABP (*r* = 0.153, *p* = 0.071, Figure 2K).

### Relationships Between Microbial Translocation and Immune Activation Markers

As microbial translocation has been linked to immune activation, we looked for correlations between the microbial translocation and the immune activation markers we had quantified. This analysis revealed that the direct marker of microbial translocation rDNA was linked to CD8+ T-cell exhaustion (*r* = 0.253, *p* = 0.003, Figure 2L) and to the endothelium activation marker sThrombomodulin (*r* = 0.239, *p* = 0.004, Figure 2M). The indirect marker of microbial translocation LBP was not only linked to CD8+ T-cell exhaustion (*r* = 0.226, *p* = 0.008, Figure 2N), but also to sThrombomodulin (*r* = 0.278, *p* = 0.002, Figure 2O), the inflammation marker CRP (*r* = 0.204, *p* = 0.017, Figure 2P) as well. The other indirect marker of microbial translocation, sCD14, correlated with the expression of the immune activation marker HLA-DR on CD4+ T-cells (*r* = 0.187, *p* = 0.028, Figure 2Q).

### Microbial Translocation Is Linked to a Specific Immune Activation Profile

**Figure 3:**
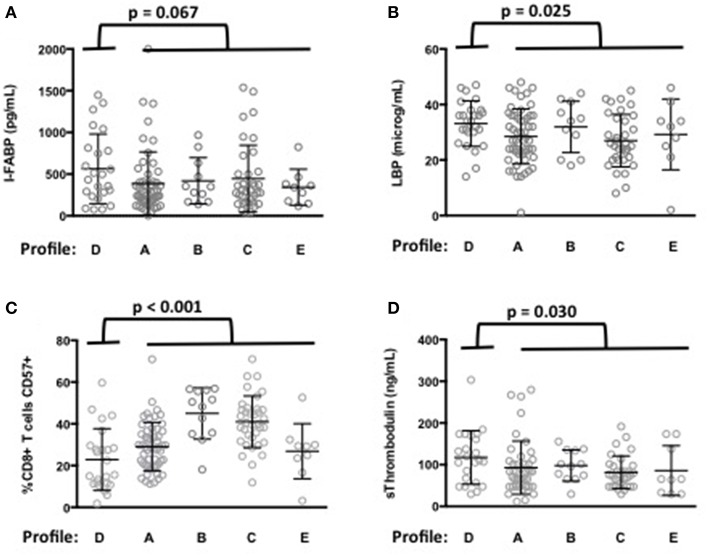
Microbial translocation markers are elevated in patients with immune activation profile D. I-FABP **(A)** and LBP **(B)** mean values and standard deviations in patients according to their immune activation profile. *p*-value of the difference for each microbial translocation marker between Profile D and the other immune activation profiles is indicated. Characterization of the immune activation profile D **(C,D)**. Differences in the percentage of CD8+ T-cells expressing CD57 **(C)** and in the level of sThrombomodulin **(D)** between profile D and the other profiles are shown.

Next, we analyzed whether the levels of microbial translocation were similar among the five immune activation profiles identified. Compared with the other patients, we observed that patients with Profile D presented a non-significant higher mean (±SEM) rDNA levels (29 ± 10 vs. 14 ± 1 copies/μL, *p* = 0.589). Their sCD14 (3.7 ± 0.2 vs. 3.3 ± 0.1 μg/mL, *p* = 0.102) and I-FABP (564 ± 85 vs. 410 ± 36 pg/mL, *p* = 0.067, Figure 3A) concentrations also tended to be higher. Moreover, their LBP levels were significantly higher (33.3 ± 1.7 vs. 28.7 ± 0.9 μg/mL, *p* = 0.025, Figure 3B), with an odds ratio (OR) per unit increase in LBP of 1.05 (95% CI, 1.01–1.11, *p* = 0.034).

Patients with Profile D were younger than the other patients (52 ± 7 vs. 57 ± 9 years, *p* = 0.003). A multivariate analysis, adjusted with age as a covariate, still revealed their risk of higher LBP (OR per unit increase in LBP of 1.06, 95% CI, 1.01–1.11, *p* = 0.030). Their CD4 nadir (148 ± 108 vs. 163 ± 104 cells/μL, *p* = 0.501) and duration of aviremia (85 ± 45 vs. 100 ± 49 months, *p* = 0.188) were not different. Although their CD8+ T-cells expressed higher levels of the activation marker CD38 (Figure 2D) they expressed lower levels of the senescence marker CD57 (23 ± 3 vs. 34 ± 1%, *p* < 0.001, Figure 3C) than the rest of the patients. Profile D was also remarkable because of a high level of mean (±SEM) sThrombomodulin (117 ± 13 vs. 89 ± 5 ng/mL, *p* = 0.030, Figure 3D).

## Discussion

We had previously described five different immune activation profiles in 120 patients with a mean age of 58 years, a mean pre-therapeutic CD4 count of 192 cells per μL, and a mean duration of infection of 17 years. It was important to assess whether the same kind of profiles could be found after having added a divergent group of patients. Here we show that adding to this cohort 20 younger individuals with a shorter period of infection and aviremia and a higher CD4 count before ART, did not disrupt the hierarchical clustering. This observation argues for the fact that our classification is robust rather than specific for a particular set of patients. Yet, this remains to be confirmed with a greater number of patients with a higher diversity.

In the present study, we found elevated microbial translocation markers in some of the virological responders we analyzed. Globally, the peripheral blood concentrations of bacterial DNA and indirect translocation markers (LBP and sCD14) were higher in infected than in non-infected individuals. It is important to note that we observed a correlation between rDNA and LBP. In contrast, we did not establish any relationship between either rDNA, LBP, sCD14, or I-FABP, and sCD163, an alternative marker for monocyte activation. This is in line with a fact we had already unraveled in the ACTIVIH study, that sCD14 and sCD163 are clustered far apart from each other in the hierarchical clustering of the activation markers (Figure 1). This further emphasizes the fact that sCD14 and sCD163 correspond to different forms of monocyte activation.

We also unveiled links between microbial translocation markers and some of the immune activation markers we measured. We noticed a link between the level of circulating bacterial DNA as well as LBP and CD8+ T-cell exhaustion and sThrombomodulin. Thrombomodulin is released from activated endothelial cells, in the course of sepsis for example (19). Therefore, sThrombomodulin is considered as an endothelial activation marker (20). Vascular endothelial cells have been reported to express the LPS receptor TLR4, particularly under proatherogenic stimuli (21). Consequently, under LPS exposure, these cells release the inflammatory cytokines IL-6 and IL-8, produce TNFα and IL-1β mRNA and express the adhesion molecules ICAM-1, VCAM, and E-selectin (21, 22). In line with the hypothesis that microbial translocation might directly activate endothelial cells, sCD14 has been shown to correlate with the markers of endovascular dysfunction, symmetric, and asymmetric dimethylarginine (23).

The main result of our work is the report of a correlation between the markers of microbial translocation LBP and I-FABP, and one profile of immune activation. Although our data do not allow to state that this link is causative, it is noteworthy that patients with Profile D present high levels of sThrombomodulin, since microbial translocation is known to induce endothelial activation (22). This argues for a causative relationship between microbial translocation and Profile D immune activation. The link between LBP and Profile D that we established strengthens the hypothesis that the immune activation profiles we observed might be fueled by specific causes. Yet we cannot exclude the possibility that, inversely, Profile D immune activation favors microbial translocation. For instance, we discovered a link between LBP and CRP, as previously observed (24). On the one hand, microbial translocation may cause inflammation, but on the other hand, inflammation may increase gut epithelium permeability and thereby microbial translocation. So there may be a bidirectional link between Profile D and microbial translocation.

As we have previously shown that a specific immune activation profile is strongly linked to a pathogenic process, insulin resistance, the present data have important consequences. Microbial translocation has been reported to be a driver of morbi-mortality, including insulin resistance (25), hypertension (26), cardiovascular disease (27), neurocognitive disorders (28), depression (29), liver disease progression (30), and non-Hodgkin lymphoma (31). Linking microbial translocation to Profile D and, in the future, eventually linking Profile D to specific morbidities, will help to specify which comorbidity microbial translocation may fuel. Early diagnosis of microbial translocation may then orientate the prevention and screening toward particular morbidities. Moreover, our observation opens the possibility to identify the pathophysiological pathways between microbial translocation and phenotypes of immune activation specific to Profile D and these phenotypes to comorbidities driven by Profile D. It will also be of interest to monitor the effect of probiotics and prebiotics tested in HIV infection (32) on Profile D. Furthermore, the influence of the dysbiosis described in HIV patients on Profile D will also have to be tested.

From a more general point of view, our observations may benefit other situations where microbial translocation occurs, including inflammatory bowel disease, pancreatitis, non-alcoholic steato-hepatitis, hepatitis B and C virus infection, graft vs. host disease, alcoholism, and aging. It would be of interest to study whether the same immune activation phenotype may be observed in these situations.

## Data Availability

The datasets generated for this study are available on request to the corresponding author.

## Ethics Statement

The studies involving human participants were reviewed and approved by Ethics Committee of the University Hospital of Montpellier. The patients/participants provided their written informed consent to participate in this study.

## Author Contributions

MY and LK contributed to the design of the flow cytometry study, acquired, analyzed, interpreted cell surface, and soluble marker data. CP contributed to the design of the study, the enrolment of patients, acquired, analyzed, and interpreted the data. RC, PP, and TV contributed to the design of the flow cytometry study, acquired, analyzed, and interpreted cell surface markers. CR, RS, AW, and PF contributed to the design of the statistical study, acquired, analyzed, and interpreted the statistical data. CM, NA, CF, VL, CB, and AS acquired, analyzed, and interpreted clinical data. CD-R, OM, and J-PL contributed to the design, analysis, and interpretation of the microbial translocation data. JR contributed to the design of the study, analyzed and interpreted the data. PC contributed to the design of the study, analyzed, interpreted data, and wrote the first draft of the manuscript. All authors revised and approved the final version.

### Conflict of Interest Statement

The authors declare that the research was conducted in the absence of any commercial or financial relationships that could be construed as a potential conflict of interest.
